# Functional transcriptomic annotation and protein–protein interaction analysis identify EZH2 and UBE2C as key upregulated proteins in ovarian cancer

**DOI:** 10.1002/cam4.1406

**Published:** 2018-03-25

**Authors:** Sandra Martínez‐Canales, Miguel López de Rodas, Miriam Nuncia‐Cantarero, Raquel Páez, Eitan Amir, Balázs Győrffy, Atanasio Pandiella, Eva María Galán‐Moya, Alberto Ocaña

**Affiliations:** ^1^ Translational Research Unit and Translational Oncology Laboratory Albacete University Hospital and Centro Regional de Investigaciones Biomedicas Castilla‐La Mancha University (CRIB‐UCLM) Albacete Spain; ^2^ Division of Medical Oncology and Hematology Princess Margaret Cancer Centre University of Toronto Toronto ON Canada; ^3^ Semmelweis University 2nd Department of Pediatrics Budapest Hungary; ^4^ Cancer Research Center and CIBERONC, CSIC Salamanca Spain

**Keywords:** Clinical outcome, druggable proteins, EZH2, Ovarian cancer, protein–protein interaction, UBE2C

## Abstract

Although early stage ovarian cancer is in most cases a curable disease, some patients relapse even with appropriate adjuvant treatment. Therefore, the identification of patient and tumor characteristics to better stratify risk and guide rational drug development is desirable. Using transcriptomic functional annotation followed by protein–protein interacting (PPI) network analyses, we identified functions that were upregulated and associated with detrimental outcome in patients with early stage ovarian cancer. Some of the identified functions included cell cycle, cell division, signal transduction/protein modification, cellular response to extracellular stimuli or transcription regulation, among others. Genes within these functions included *AURKA, AURKB, CDK1, BIRC5,* or *CHEK1* among others. Of note, the histone‐lysine N‐methyltransferase (*EZH2*) and the ubiquitin‐conjugating enzyme E2C (*UBE2C*) genes were found to be upregulated and amplified in 10% and 6% of tumors, respectively. Of note, EZH2 and UBE2C were identified as principal interacting proteins of druggable networks. In conclusion, we describe a set of genes overexpressed in ovarian cancer with potential for therapeutic intervention including *EZH2* and *UBE2C*.

## Introduction

Disseminated ovarian cancer is an incurable disease 1. However, if diagnosed in its early stage, resection and adjuvant chemotherapy can reduce the probability of the tumor to relapse and spread 2. Unfortunately, some patients with early stage ovarian cancer (mainly stage 1 and 2) are still at high risk of relapse, even after being treated with adequate surgical and adjuvant chemotherapy 2. In this context, the identification of patients who have high risk of recurrence is desirable as it can influence adjuvant treatment and guide future drug development.

Similar to other cancers, in ovarian cancer, different molecular mechanisms are responsible for cancer initiation and progression. Uncontrolled proliferation, migration, evasion from immunological regulation, or the capacity to generate new vessels are, among others, oncogenic hallmarks of ovarian cancer 3. Of note, agents that mitigate these functions, such as antimitotic chemotherapies, DNA damaging agents or anti‐angiogenic compounds, have reached the clinical practice 3, 4. Among agents that target classical deregulated functions such as cell division or proliferation, novel vulnerabilities with potential for therapeutic capacity are under evaluation, including protein modifications or epigenetic events. New drugs targeting the proteasome, ubiquitination, or bromodomains are currently under evaluation in several solid tumors 5.

In this context, it will be desirable to identify biological functions that are characteristically deregulated in ovarian cancer at a transcriptomic and proteomic level. Genomic signatures and protein–protein interacting networks could be used to select patients with higher risk of relapse in the long term. Furthermore, molecular elements involved in these biological functions could be potentially druggable, opening the door to evaluate new compounds against these alterations in the clinical setting. With this approach in mind, we have described genes and gene signatures associated with mitosis that predicted poor outcome specifically in patients with early stage ovarian tumors 6. However, we envision that an analysis based of functional genomics and protein–protein interactions could provide more robust prediction outcome in ovarian cancers, and a more general overview of the biological characteristics of this disease.

In this project using an *in silico* approach using public transcriptomic data, we identified deregulated functions in early stage ovarian cancer that were associated with worse outcome. Expression of some of these signatures identified patients at a higher risk. A protein–protein interaction analysis revealed hubs of proteins with oncogenic implications that could be inhibited pharmacologically. Of note, a relevant finding was the identification of the histone‐lysine N‐methyltransferase EZH2, and the ubiquitin‐conjugating enzyme E2C as key upregulated interacting proteins. In addition, these proteins were amplified in 10% and 6% of the ovarian tumors. The data presented opens the door to the further assessment of these signatures in clinical studies, and for the evaluation of novel therapies against the mentioned proteins or pathways.

## Material and Methods

### Transcriptomic and gene expression analyses

To identify differences at a transcriptomic level, we used a public dataset (GEO DataSet accession number: GSE14407) of mRNA levels from twelve isolated ovarian epithelial cell lines and twelve isolated serous ovarian cancer epithelial (CEPI) cells. Affymetrix CEL files were downloaded and analyzed with Affymetrix Transcriptome Analysis Console 3.0. Differential gene expression profile for both groups was performed using a minimum fourfold change. Oncomine^™^ Platform was used to confirm the GEO DataSet findings (https://www.oncomine.org/resource/login.html).

### Evaluation of clinical outcome

The publicly available Kaplan–Meier (KM) Plotter Online Tool (http://kmplot.com/analysis/) was used to evaluate the relationship between gene expression levels and patient's clinical outcome in early stage ovarian cancer (stage I and II). Only genes significantly associated with detrimental outcome (Hazard Ratio ≥1 and *P*‐value ≤0.05) were used for subsequent analysis (*n *= 131). This tool was also used to determine progression‐free survival (PFS) and overall survival (OS) in functional combined analyses. All the analyses were performed independently by two authors (SMC and MLR) and reviewed by a third author (EMGM) (Accession date January 8th 2018). No discrepancies were observed.

### Protein–protein interactions maps and functional evaluation

Using the String Online Tool (http://www.string-db.org), we constructed the interactome. The PPI map was based on the list of genes associated with poor PFS. Proteins showing less than two interactions were not considered. Subsequently, we performed a functional screening using Ensembl (http://www.ensembl.org), and Gene Ontology (GO) by biological function.

### Selection of potential drug candidates

We used information from Selleckchem (http://www.selleckchem.com) and Genecards (http://www.genecards.org) to select potentially druggable genes. Then, as described above, we used the STRING tool to build the druggable ovarian cancer interactome. Based on interacting groups, we divided the PPI map in three functional clusters: cell cycle (*n *= 19), DNA damage (*n *= 4), and angiogenesis (*n *= 3). PPI hubs proteins were determined as those with a higher number of interactions than the average (Edges ≥17.2).

### Identification of molecular alterations

We used data contained at cBioportal (http://www.cbioportal.org; TCGA Ovarian Serous Cystadenocarcinoma, *n *= 603) to identify potential copy number alterations (amplification or deletion), and the presence of mutations in the identified genes.

## Results

### Selection of deregulated genes and functional analyses

**Figure 1:**
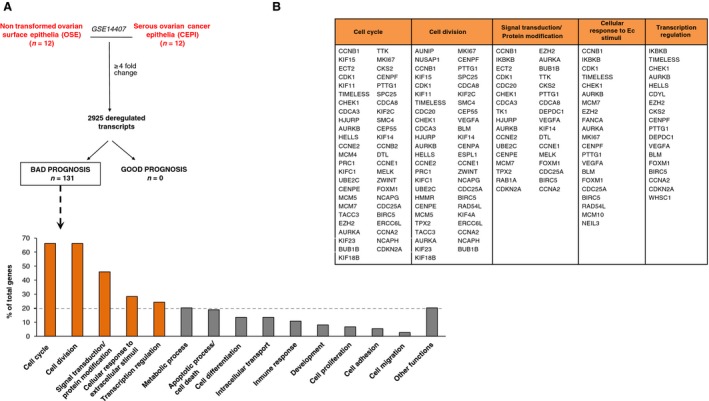
Transcriptomic analyses comparing isolated serous ovarian cancer epithelial (CEPI) cells with isolated ovarian epithelial cells. (A) Identification of deregulated genes (fold change ≥4) which are associated with bad prognosis in CEPI. (B) Functional enrichment analyses identify cell cycle, cell division, signal transduction/protein modification, cellular response to extracellular stimuli and transcription regulation, as the most altered functions in CEPI.

To identify deregulated functions in ovarian cancer cells, we used public transcriptomic data (GSE14407), to compare isolated serous ovarian cancer epithelial (CEPI) cells with isolated ovarian epithelial cell lines. Using a minimum fold change of four, we identified 2925 genes of which 131 were associated with poor clinical outcome (Fig. 1A and Table 1). The upregulation of the genes was confirmed using data from human samples contained at Oncomine (Table 1). Protein–protein interaction network showed 130 nodes and a cluster coefficient of 0.62 (Fig. S1).

**Table 1:** List of deregulated genes associated with bad prognosis

Probe ID	Transcript ID	Gene symbol	AFFYMETRIX		ONCOMINE		KMPLOTTER	
							PFS	
			Fold change	*P*‐Value ANOVA	Fold change	*P*‐Value	HR	*P*‐Value
211767_at	g13543688	GINS4	4.01	0.002866	2.404	1.11E‐05	2.87 (1.54–5.35)	0.0005
228729_at	Hs.23960.0	CCNB1	4.03	8.24E‐07	6.152	5.33E‐06	2.85 (1.3–6.22)	0.0062
1569241_a_at	Hs2.149839.1	ZNF93	4.06	0.004685	2.296	6.40E‐06	2.41 (1.14–5.08)	0.0176
205869_at	g4506144	PRSS1	4.06	0.000394	2.008	2.12E‐07	1.91 (1.06–3.45)	0.0296
213100_at	Hs.13350.0	UNC5B	4.07	0.000451	1.351	0.006	1.82 (1.01–3.3)	0.0432
216615_s_at	Hs.2142.1	HTR3A	4.11	0.001305	3.505	4.76E‐16	2.23 (1.19–4.15)	0.0098
40020_at	4858618_RC	CELSR3	4.13	9.48E‐08	1.502	7.89E‐07	3.86 (1.98–7.54)	0
219306_at	g9910265	KIF15	4.17	0.000147	3.696	2.29E‐08	2.88 (1.54–5.38)	0.0005
209342_s_at	g4185274	IKBKB	4.17	0.007424	1.31	2.12E‐04	2.44 (1.31–4.56)	0.0038
213759_at	Hs.111554.1	ARL4C	4.21	0.008012	3.624	8.81E‐06	1.89 (1.06–3.39)	0.0289
206134_at	g7657318	ADAMDEC1	4.21	0.003324	1.87	0.012	1.88 (1.04–3.39)	0.0337
219787_s_at	g8922431	ECT2	4.23	0.000033	10.209	2.81E‐08	2.62 (1.42–4.83)	0.0014
210559_s_at	g3126638	CDK1	4.23	0.000803	7.317	4.85E‐07	1.8 (1.01–3.23)	0.0447
204444_at	g13699823	KIF11	4.25	0.000272	5.467	7.52E‐07	2.73 (1.48–5.03)	0.0008
209053_s_at	Hs.110457.3	WHSC1	4.25	0.000004	3.805	3.67E‐10	3.03 (1.57–5.84)	0.0005
209198_s_at	g13279139	SYT11	4.27	0.000005	2.974	1.43E‐07	3.4 (1.81–6.39)	0.0001
207156_at	g10800131	HIST1H2AG	4.3	0.006192	1.554	5.81E‐06	1.96 (1.1–3.5)	0.0201
205544_s_at	g4503026	CR2	4.34	0.027892	1.428	6.37E‐06	3.37 (1.74–6.53)	0.0001
203046_s_at	g4507506	TIMELESS	4.36	4.84E‐07	3.434	2.30E‐09	1.86 (1.03–3.37)	0.0365
202870_s_at	g4557436	CDC20	4.41	0.000004	11.259	2.44E‐06	3.87 (2.01–7.46)	0
202860_at	g7662151	DENND4B	4.43	0.000019	1.563	3.19E‐04	3.3 (1.71–6.37)	0.0002
214933_at	Hs.96253.2	CACNA1A	4.45	0.000001	2.563	1.47E‐05	2.6 (1.39–4.85)	0.0018
210587_at	g13477368	INHBE	4.47	0.042977	1.32	7.38E‐05	2.22 (1.2–4.09)	0.0089
214005_at	Hs.77719.1	GGCX	4.5	2.02E‐08	1.837	2.04E‐04	2.45 (1.35–4.46)	0.0025
205660_at	g11321576	OASL	4.53	0.006553	2.449	9.95E‐05	2.06 (1.13–3.78)	0.0165
219454_at	g13124887	EGFL6	4.54	0.000439	2.582	5.00E‐03	2.34 (1.27–4.31)	0.0049
212816_s_at	Hs.84152.2	CBS	4.55	0.000561	2.658	1.00E‐03	2.06 (1.14–3.74)	0.0146
205394_at	g4502802	CHEK1	4.6	0.000004	4.147	2.43E‐07	2.04 (1.13–3.66)	0.015
221436_s_at	g13876383	CDCA3	4.65	0.001047	4.847	1.30E‐09	2.09 (1.15–3.77)	0.0128
207109_at	g7657408	POU2F3	4.66	0.024312	1.741	4.43E‐04	2.81 (1.53–5.18)	0.0005
202219_at	g5032096	SLC6A8	4.68	0.000195	1.94	1.76E‐07	2.17 (1.18–4)	0.0108
217025_s_at	Hs.89434.1	DBN1	4.69	0.007175	2.141	1.84E‐06	2.11 (1.13–3.93)	0.0163
202338_at	g4507518	TK1	4.73	0.00005	4.968	1.55E‐08	2.07 (1.13–3.77)	0.0156
222251_s_at	Hs.28906.1	GMEB2	4.81	0.004936	1.339	3.27E‐04	5.5 (2.57–11.76)	0
210697_at	g4454677	ZNF257	4.82	0.001284	1.673	1.66E‐05	2.06 (1.15–3.7)	0.0135
214339_s_at	Hs.86575.2	MAP4K1	4.87	0.000074	1.866	1.13E‐05	2.07 (1.13–3.79)	0.0154
203022_at	g5454009	RNASEH2A	4.94	0.000147	2.785	1.11E‐06	2.06 (1.13–3.76)	0.016
206280_at	g4826670	CDH18	4.96	0.006096	1.396	0.004	2.05 (1.14–3.7)	0.0143
211343_s_at	g180828	COL13A1	5	0.000822	1.318	3.17E‐04	2.05 (1.13–3.75)	0.0165
206513_at	g4757733	AIM2	5.02	0.007612	1.547	6.09E‐04	2.73 (1.46–5.11)	0.0011
204994_at	g11342663	MX2	5.03	0.001647	3.916	3.32E‐04	2.2 (1.19–4.06)	0.0098
205163_at	g7019426	MYLPF	5.04	0.000789	1.397	4.94E‐06	1.97 (1.1–3.54)	0.0201
218726_at	g8922180	HJURP	5.07	0.010918	5.547	2.20E‐09	1.95 (1.08–3.5)	0.023
239219_at	Hs.221197.0	AURKB	5.1	0.001028	2.818	2.34E‐05	2.22 (1.04–4.76)	0.0353
202575_at	g6382069	CRABP2	5.27	0.000004	3.216	9.08E‐05	2.08 (1.16–3.74)	0.0124
35160_at	4870487_RC	LDB1	5.29	0.00032	1.5	1.00E‐03	2.35 (1.27–4.32)	0.0048
212556_at	Hs.239784.0	SCRIB	5.31	5.24E‐07	2.578	8.65E‐07	2 (1.1–3.65)	0.021
203439_s_at	g12653744	STC2	5.31	0.001003	2.509	1.53E‐06	1.85 (1.03–3.35)	0.0379
234040_at	Hs.287543.0	HELLS	5.35	0.004925	2.352	3.86E‐05	2.39 (1.11–5.11)	0.0209
221125_s_at	g7657250	KCNMB3	5.47	0.000016	1.637	2.06E‐06	2.03 (1.11–3.71)	0.0183
205569_at	g7657660	LAMP3	5.48	0.040774	3.979	3.15E‐04	3.56 (1.85–6.85)	0.0001
213520_at	Hs.31442.0	RECQL4	5.48	0.00012	1.358	0.002	2.61 (1.4–4.87)	0.0018
205034_at	g4757931	CCNE2	5.49	0.00002	1.344	2.00E‐03	2.29 (1.27–4.11)	0.0046
222037_at	Hs.154443.1	MCM4	5.52	0.013214	4.726	8.00E‐08	2.9 (1.55–5.41)	0.0005
218494_s_at	g13236503	SLC2A4RG	5.64	0.000878	2.118	5.86E‐06	2.48 (1.33–4.62)	0.0031
212235_at	Hs.301685.0	PLXND1	5.64	0.000117	1.496	4.67E‐04	2.05 (1.13–3.71)	0.0152
218296_x_at	g8922469	MSTO1	5.72	0.000026	1.381	0.018	1.83 (1–3.33)	0.0458
218009_s_at	g4506038	PRC1	5.74	9.19E‐07	7.214	5.32E‐08	2.85 (1.53–5.32)	0.0006
209680_s_at	g12653842	KIFC1	5.78	0.000129	3.845	3.64E‐08	2.33 (1.28–4.24)	0.0046
202954_at	g5902145	UBE2C	5.81	2.13E‐08	10.184	2.24E‐07	3.03 (1.62–5.66)	0.0003
205240_at	g9558734	GPSM2	6.01	0.000545	3.965	2.97E‐08	1.81 (1.01–3.25)	0.0435
209262_s_at	g12803666	NR2F6	6.05	2.41E‐07	1.61	2.18E‐05	2.26 (1.23–4.17)	0.0071
203632_s_at	g7706450	GPRC5B	6.12	0.000022	1.672	0.004	2.22 (1.21–4.04)	0.0078
207165_at	g7108350	HMMR	6.14	0.000011	3.819	1.48E‐10	2.35 (1.3–4.25)	0.0037
205046_at	g4502780	CENPE	6.16	0.00057	2.711	1.59E‐07	2.55 (1.36–4.75)	0.0024
208394_x_at	g13259505	ESM1	6.2	0.000021	1.496	0.009	2.05 (1.13–3.71)	0.0152
216237_s_at	Hs.77171.1	MCM5	6.22	0.001843	1.795	7.33E‐05	2.33 (1.25–4.34)	0.0063
205449_at	g9558738	SAC3D1	6.31	0.000029	1.891	3.53E‐05	2.19 (1.2–4)	0.0086
203099_s_at	g4558755	CDYL	6.33	0.000004	1.889	4.26E‐05	2.1 (1.15–3.83)	0.013
210983_s_at	g12751125	MCM7	6.48	0.008102	3.523	2.31E‐07	2.23 (1.21–4.1)	0.0084
210052_s_at	g6073830	TPX2	6.5	2.90E‐08	13.887	1.65E‐07	2.55 (1.38–4.69)	0.0019
225846_at	Hs.24743.1	ESRP1	6.53	0.000005	2.135	2.68E‐04	2.3 (1.04–5.06)	0.0335
218308_at	g5454101	TACC3	6.54	0.000462	4.047	9.61E‐06	4.1 (2.04–8.24)	0
239570_at	Hs.144137.0	RAB1A	6.76	0.000581	1.31	3.07E‐04	2.48 (1.1–5.6)	0.0242
203358_s_at	g4758323	EZH2	6.84	0.000002	6.584	1.44E‐06	3.63 (1.93–6.8)	0
203806_s_at	g4503654	FANCA	6.87	0.00001	1.793	7.55E‐05	2.69 (1.42–5.08)	0.0016
219502_at	g8922721	NEIL3	6.91	0.000004	1.519	1.08E‐05	2.36 (1.3–4.28)	0.0035
208079_s_at	g4507278	AURKA	7	3.64E‐08	6.504	6.53E‐08	2.95 (1.6–5.45)	0.0003
204709_s_at	g13699831	KIF23	7	0.000061	4.68	2.17E‐06	2.7 (1.48–4.95)	0.0008
203755_at	g5729749	BUB1B	7.09	6.83E‐10	8.04	2.56E‐07	2.86 (1.55–5.29)	0.0004
222039_at	Hs.274448.1	KIF18B	7.2	6.26E‐09	2.135	4.91E‐06	2.4 (1.31–4.37)	0.0032
204822_at	g4507718	TTK	7.21	7.51E‐07	15.153	2.06E‐09	2.52 (1.38–4.61)	0.0019
212023_s_at	Hs.80976.1	MKI67	7.25	1.04E‐07	4.023	5.17E‐10	1.94 (1.07–3.51)	0.0256
204170_s_at	g4502858	CKS2	7.39	6.82E‐08	5.956	3.85E‐05	2.06 (1.14–3.73)	0.0147
207183_at	g5453665	GPR19	7.43	0.000063	2.901	8.95E‐09	3.07 (1.64–5.72)	0.0002
207828_s_at	g4885132	CENPF	7.52	0.000002	3.811	1.75E‐06	2.64 (1.43–4.87)	0.0013
206157_at	g4506332	PTX3	7.56	0.000006	2.79	0.004	2.93 (1.6–5.37)	0.0003
218039_at	g7705950	NUSAP1	7.63	5.89E‐09	9.731	7.45E‐07	2.08 (1.16–3.75)	0.0123
203554_x_at	g11038651	PTTG1	7.68	0.000002	5.99	1.80E‐05	3.34 (1.76–6.34)	0.0001
209891_at	g9963834	SPC25	7.73	0.000027	2.928	9.73E‐24	2.45 (1.34–4.47)	0.0026
221520_s_at	g12804484	CDCA8	7.78	0.000076	3.705	5.44E‐07	2.63 (1.41–4.91)	0.0016
218755_at	g5032012	KIF20A	7.9	1.87E‐08	9.021	9.21E‐08	2.56 (1.37–4.78)	0.0021
201761_at	g13699869	MTHFD2	7.92	0.000004	3.82	1.20E‐04	2.68 (1.45–4.94)	0.001
204649_at	g4885624	TROAP	7.95	0.000014	3.096	5.11E‐08	2.83 (1.49–5.35)	0.0008
209408_at	g1695881	KIF2C	8.01	2.31E‐08	2.834	6.75E‐11	2.43 (1.33–4.44)	0.003
201663_s_at	g4885112	SMC4	8.12	0.008518	7.44	9.32E‐09	2.55 (1.38–4.71)	0.0019
218542_at	g8922501	CEP55	8.28	0.000158	8.075	1.50E‐08	1.89 (1.05–3.4)	0.0304
222958_s_at	Hs.133260.0	DEPDC1	8.47	0.000182	3.833	2.12E‐07	2.61 (1.19–5.7)	0.0127
222008_at	Hs.154850.0	COL9A1	8.48	0.000545	1.946	2.30E‐16	1.93 (1.08–3.47)	0.0249
210512_s_at	g3719220	VEGFA	8.51	6.97E‐09	2.741	1.17E‐07	3.37 (1.75–6.48)	0.0001
205733_at	g4557364	BLM	8.53	0.000002	2.88	3.59E‐06	1.99 (1.1–3.59)	0.0205
236641_at	Hs.116649.0	KIF14	8.88	0.000311	3.139	5.27E‐06	2.28 (1.01–5.13)	0.0414
204962_s_at	g4585861	CENPA	8.9	0.000014	11.775	2.63E‐09	2.48 (1.35–4.57)	0.0026
202705_at	g10938017	CCNB2	9.15	1.13E‐07	10.154	1.59E‐06	1.87 (1.04–3.37)	0.0329
218585_s_at	g7705575	DTL	9.2	2.49E‐10	6.089	1.58E‐07	1.89 (1.06–3.38)	0.0289
38158_at	4852842_RC	ESPL1	9.2	8.96E‐09	4.341	6.11E‐07	3.19 (1.69–6.04)	0.0002
213523_at	Hs.9700.0	CCNE1	9.36	2.03E‐07	7.062	8.28E‐09	1.84 (1.02–3.33)	0.0407
222946_s_at	g12652906	AUNIP	9.41	0.00175	2.956	7.47E‐08	2.11 (0.99–4.52)	0.0485
213075_at	Hs.94795.0	OLFML2A	9.44	0.000481	1.423	3.00E‐03	1.86 (1.04–3.34)	0.0348
204825_at	g7661973	MELK	9.59	0.000007	10.6	2.98E‐07	1.95 (1.08–3.5)	0.0233
212563_at	Hs.30736.0	BOP1	9.61	0.000165	1.669	3.35E‐06	2.03 (1.11–3.69)	0.0186
204026_s_at	g6857828	ZWINT	9.92	1.65E‐09	7.001	1.71E‐05	2.05 (1.14–3.7)	0.015
202580_x_at	g11386144	FOXM1	10.06	0.000022	5.982	8.64E‐09	3.03 (1.6–5.73)	0.0003
205694_at	g4507756	TYRP1	10.15	0.000772	1.624	2.09E‐34	1.79 (1–3.23)	0.0486
204584_at	Hs.1757.0	L1CAM	10.27	0.000008	3.985	7.02E‐15	3.02 (1.63–5.58)	0.0002
218662_s_at	g11641252	NCAPG	10.35	4.18E‐08	3.207	2.13E‐10	3.28 (1.76–6.14)	0.0001
204695_at	Hs.1634.0	CDC25A	10.39	0.000002	2.633	2.49E‐05	2.39 (1.31–4.34)	0.0033
212807_s_at	Hs.281706.1	SORT1	10.54	4.63E‐07	1.977	9.60E‐06	2.05 (1.12–3.75)	0.0172
202094_at	Hs.1578.0	BIRC5	10.82	0.000018	4.83	2.20E‐10	2.85 (1.53–5.31)	0.0006
204558_at	g4506396	RAD54L	11.24	0.000856	2.09	6.38E‐06	2.85 (1.53–5.32)	0.0006
218355_at	g7305204	KIF4A	11.53	5.42E‐08	2.359	1.00E‐03	2.82 (1.51–5.27)	0.0007
219650_at	g8923111	ERCC6L	12.29	6.30E‐07	2.245	8.13E‐06	2.24 (1.24–4.04)	0.0063
204437_s_at	g9257206	FOLR1	12.72	0.000017	1.696	2.00E‐03	2.05 (1.13–3.7)	0.0155
203418_at	g4502612	CCNA2	13.14	0.00196	4.795	2.28E‐07	1.82 (1.02–3.26)	0.0403
205242_at	g5453576	CXCL13	16.29	0.0001	2.091	2.94E‐08	1.83 (1.02–3.28)	0.0411
205572_at	g4557314	ANGPT2	19.81	0.000006	1.312	0.029	2.02 (1.12–3.63)	0.0164
212949_at	Hs.1192.0	NCAPH	19.94	7.17E‐07	2.497	1.51E‐06	3.06 (1.62–5.78)	0.0003
206772_at	g4826953	PTH2R	21.94	0.000027	5.579	8.58E‐10	3.14 (1.66–5.97)	0.0002
222962_s_at	g11527601	MCM10	29.44	6.72E‐09	2.718	1.28E‐07	2.33 (1.06–5.1)	0.0296
207039_at	g4502748	CDKN2A	45.1	0.000022	6.481	5.60E‐14	2.01 (1.11–3.63)	0.0186
206373_at	g4507970	ZIC1	99.77	2.13E‐08	3.712	8.24E‐07	2.19 (1.22–3.93)	0.0073

### Functional gene signatures associated with poor outcome

Functional annotation of the identified genes demonstrated several altered functions (Fig. 1A and B). By selecting those more represented (with a more than 20% of total genes expression), we identified cell cycle, cell division, signal transduction/protein modification, cellular response to extracellular (EC) stimuli, and transcription regulation. Table S1 provides detailed information of all functions and genes included within each function.

**Figure 2:**
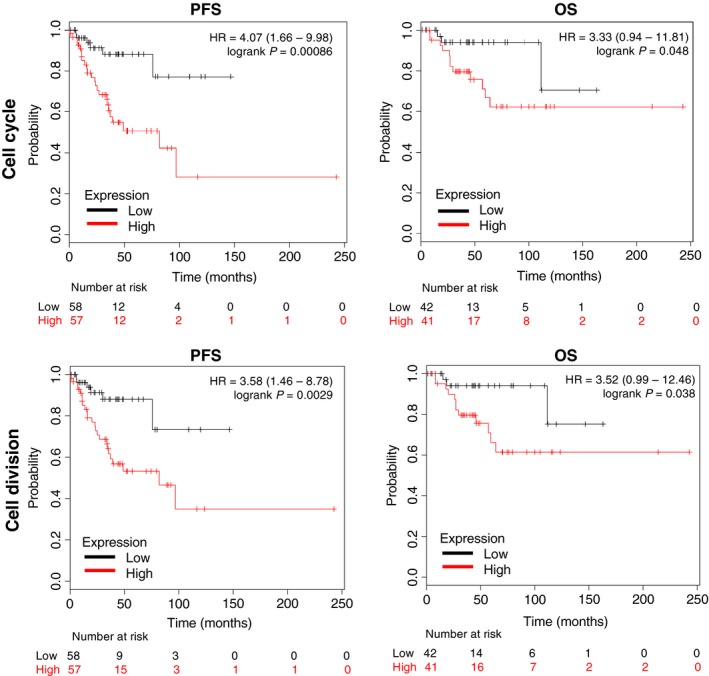
Association with progression‐free survival (PFS) and overall survival (OS) in stage I and II ovarian cancer of gene sets included in the cell cycle and cell division function.

**Figure 3:**
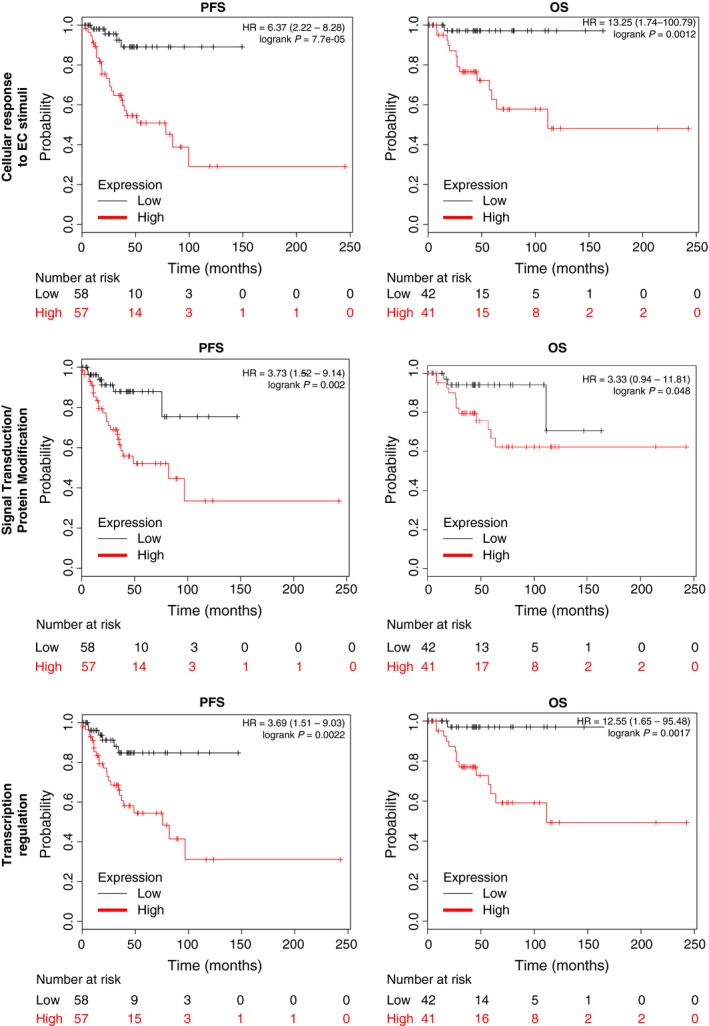
Association with progression‐free survival (PFS) and overall survival (OS) in stage I and II ovarian cancer of gene sets included in the cellular response to extracellular (EC) stimuli, signal transduction/protein modification and transcription regulation function.

Using the KM Plotter online tool, we explored the association with clinical outcome of genes within each function. We did so to observe the role of each group with clinical prognosis. Genes within the cell cycle and cell division were associated with detrimental PFS and OS (PFS: HR = 4.07 (95% CI 1.66–9.98), *P* = 0.00086 and OS: HR = 3.33 (95% CI 0.94–11.81), *P* = 0.048 for cell cycle and PFS: HR = 3.58 (95% CI 1.46–8.78), *P* = 0.0029 and OS HR =  3.52 (95% CI 0.99–12.46), *P* = 0.038, for cell division) (Fig. 2). Results in the same range were observed for signal transduction/protein modification (PFS HR = 3.73 (95% CI 1.52–9.14), *P* = 0.002 and OS HR =  3.33 (95% CI 0.94–11.81), *P* = 0.048) and for transcription regulation PFS data (PFS: HR = 3.69 (95% CI 1.51–9.03), *P* = 0.0022). Interestingly, a poorer outcome for OS was found for this latter group (OS: HR = 12.55 (95% CI 1.65–95.48), *P* = 0.0017) (Fig. 3). Finally, the group of genes within the cellular response to EC stimuli function showed the worse outcome for both PFS and OS (PFS: HR = 6.37 (95% CI 2.22–18.28), *P* = 7.7e‐05 and OS: HR = 13.25 (95% CI 1.74–100.79), (Fig. 3).

### Druggable opportunities within the identified functions

**Figure 4:**
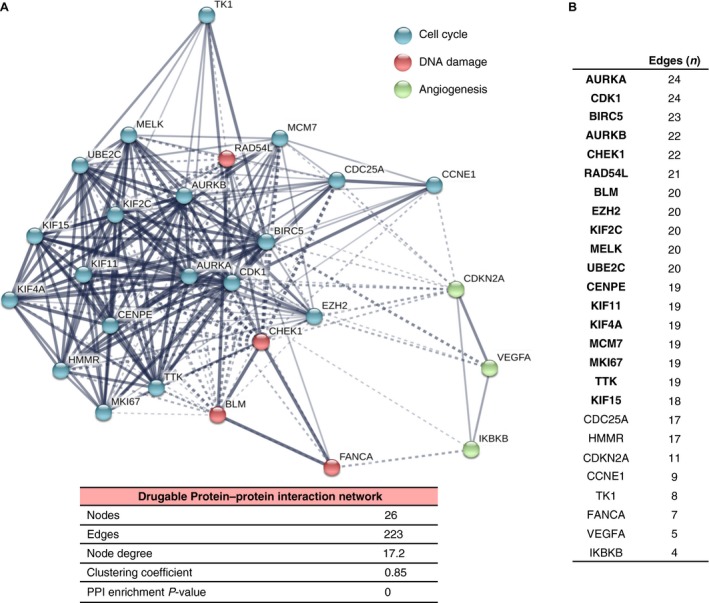
Protein–protein interaction (PPI) map of the 26 potential druggable targets. (A) Potentially druggable targets were used to construct a PPI network using the online tool STRING. Blue nodes represent proteins involved in cell cycle. Red and green nodes represent proteins associated with DNA damage and angiogenesis, respectively. The nodes indicate proteins coded by the identified druggable targets and edges indicate the number of interactions. The number of average interactions per node is represented by the node degree. The clustering coefficient indicates the average node density of the map. (B) List of hub proteins according the number of interactions (edges) in the druggable PPI network.

The description of functional signatures has the advantage of identifying relevant molecular alterations that have a potential oncogenic role in this disease, and therefore are susceptible to be inhibited. To get insights into potential therapies for those patients harboring these signatures, we used the drug gene interaction database available in Genecards and confirmed by other sources as described in Material and Methods. We therefore selected 26 genes that could potentially be inhibited pharmacologically (Table S2). We next used the proteins coded by these genes to build a protein–protein interaction network. We found 223 interactions (edges) linking 26 proteins (nodes). As expected, the clustering coefficient in this druggable network was high (0.85), confirming that most of the proteins act as a functional unit. We identified three different functional clusters with special affinity: Cell cycle (*n *= 19 genes), DNA damage (*n *= 4 genes), and angiogenesis (*n *= 3 genes) (Fig. 4A). Of note, DNA damage was included as part of the cellular response to EC stimuli in our initial functional annotation, and angiogenesis was one of the functions identified in the functional annotation studies, although was less represented (Table S1). These results suggest an important role of this in the druggable PPI. Next, based on the number of interactions, we selected the hub proteins of the interactome, defined as those with a higher number of interactions than the average (Edges ≥17.2; *n *= 18) (Fig. 4B).

Some of the genes identified here have been described previously in ovarian cancer as deregulated, including *AURKA, AURKB, CDK1, BIRC5,* and *CHEK1* among others 6. Of note, the histone‐lysine N‐methyltransferase EZH2 is a novel epigenetic target not previously described, and the ubiquitin‐conjugating enzyme E2C (UBE2C), which belongs to the ubiquitin ligase family of enzymes is also a potentially druggable protein with limited evaluation in ovarian cancer. Interestingly, these two genes strongly associate with worse prognosis for OS (Table S3)

### Molecular alterations in the identified signatures

To complete our study, we used the cancer genomics database (cBioportal 7) to obtain information about copy number alterations or mutations of the identified druggable genes. Most of genes that code for the identified druggable hubs were found to be amplified in ovarian cancer (Table 2). Of note, the new potential targets *EZH2* and *UBE2C* were amplified in around 10% and 6% of ovarian cancers, respectively. Deletions and mutations were present at a very low frequency. Amplifications of other genes such as *RAD54L, AURKA, KIF2C,* or *BIRC5* were also observed.

**Table 2:** Molecular alterations of the identified hub proteins

311 Ovarian serous cystadenocarcinoma samples			
Gene Name	Amplification	Deletion	Mutation
**EZH2**	10.30%	0.30%	–
RAD54L	9.00%	–	0.60%
AURKA	8.70%	–	–
KIF2C	6.40%	–	0.30%
BIRC5	6.10%	0.60%	–
**UBE2C**	5.80%	–	–
BLM	5.50%	0.30%	1.30%
CHEK1	3.90%	0.60%	–
MKI67	3.50%	1.00%	1.30%
MCM7	3.20%	–	–
KIF4A	1.90%	0.30%	0.60%
CDK1	1.90%	–	0.60%
TTK	1.60%	0.30%	0.60%
MELK	1.30%	–	0.60%
KIF15	1.00%	0.30%	0.30%
CENPE	0.60%	1.30%	0.60%
AURKB	0.60%	0.60%	–
KIF11	0.60%	0.30%	–

## Discussion

In the present article, we describe functional gene signatures and PPI networks associated with adverse outcome in early stage ovarian cancer. These signatures and interacting protein networks provide information about druggable opportunities that could be validated preclinically.

As ovarian cancer is an incurable disease, the identification of oncogenic functions and protein interacting networks associated with detrimental outcome is expected to improve the therapeutic landscape of this disease. In early stage ovarian cancer, the identification of patients with worse outcome is even more relevant as it may help in the selection of patients for additional adjuvant therapy, and even guide the evaluation of novel therapies.

In our study, we have identified five functions linked with detrimental PFS and OS in early stage ovarian cancer. Within cell cycle and cell division, we found genes such as *AURKA, AURKB, CDK1, BIRC5,* and *CHEK1* that are associated with control of mitosis and cell cycle regulation 8. Of note, some of these genes have been reported previously to be linked with detrimental outcome 6. Inhibitors against proteins coded by these genes, such as AURKA and B or CHEK1, are currently in clinical development, so our findings provide support for the specific development of those agents in ovarian cancer.

An interesting finding was the identification of protein modifications and transcription regulation as upregulated functions. Protein modification and degradation is a vulnerability of tumor cells as has been demonstrated by the clinical activity of proteasome inhibitors in some hematological malignancies 9, 10. Ubiquitination is a necessary pathway to target proteins for degradation 11. The ubiquitin‐conjugating enzyme E2C is required for the destruction of mitotic cyclins and for cell cycle progression 12. UBE2C has been found to be overexpressed in esophageal squamous cell carcinoma playing a role in cancer progression 13, 14, as well as, in other tumor types such as nonsmall cell lung cancer 15. However, there are no published data regarding the role of this protein in ovarian cancer. As this family of proteins can be inhibited pharmacologically 11, the study of such agents in ovarian cancer is warranted.

Other relevant findings include the identification of EZH2 as upregulated and involved in the PPI network. EZH2 has been associated with epithelial to mesenchymal transition in ovarian cancers 16. Of note, EZH2 inhibitors seem to be particularly active in malignant rhabdoid tumors, which are deficient in the Switch/Sucrose NonFermentable (SWI/SNF) chromatin remodeling complexes INI1 (SMARCB1). Of interest, a subgroup of ovarian tumors has a similar phenotype and has shown responses to inhibitors of this complex 17. In our study, we observe that EZH2 is a relevant component of the PPI network therefore confirming a potentially druggable vulnerability. Of note, drugs such as tazemetostat, a potent and selective EZH2 inhibitor is currently in phase II testing 18. Other molecular alteration includes *RAD54L* that is amplified in 9% of patients. The protein associated by this gene is involved in the homologous recombination repair of DNA double‐strand breaks 19. Finally, genes such as *KIF2C* or *AURKA* are involved in mitotic formation and chromosome segregation 20.

Our analysis highlights several druggable functions in early stage ovarian cancer for which new agents are currently in preclinical or clinical evaluation. However, we should acknowledge that our study has some limitations. This is an *in silico* analysis that need confirmatory studies using human samples. In addition, functional assessment has the limitation for the redundancy of functions, as many genes can be classified in many different annotations. Finally, there are limitations for the existed software that help identifying druggable opportunities mainly for redundancy.

**Figure 5:**
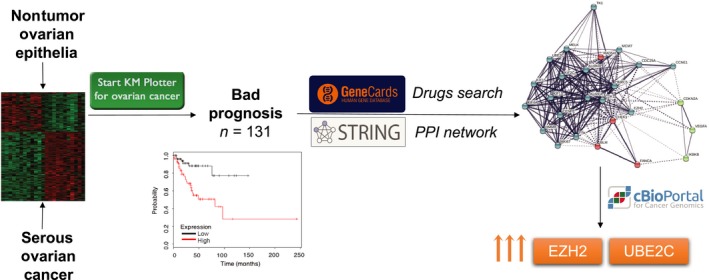
Study graphical abstract.

In conclusion, we have identified biological functions and PPI networks that are prognostic in early stage ovarian cancer and may guide future drug development (Fig. 5). Some of the identified genes such as *EZH2* or *UBE2C* have not been described previously in ovarian cancer but are amplified, linked with detrimental prognosis and potentially druggable, and warrant preclinical and clinical assessment.

## Conflict of Interest

None declared.

## Supporting information


**Table S1.** Functional classification of the deregulated genes.

**Table S2.** List of potentially druggable genes.

**Table S3.** Association with progression free survival (PFS) and overall survival (OS) of the identified hub proteins.

**Figure S1.** Protein‐protein interaction network of the 130 deregulated genes associated with detrimental prognosis.

## References

[1] Wilson, M. K. , E. Pujade‐Lauraine , D. Aoki , M. R. Mirza , D. Lorusso , A. M. Oza , A. Bois , I. Vergote , A. Reuss , M. Bacon , and M. Friedlander . 2017 Fifth Ovarian cancer consensus conference of the gynecologic cancer intergroup: recurrent disease. Ann. Oncol. 28:727–732. 27993805 10.1093/annonc/mdw663 PMC6246494

[2] Karam, A. , J. A. Ledermann , J. W. Kim , J. Sehouli , K. Lu , C. Gourley , N. Katsumata , R. A. Burger , B. H. Nam , M. Bacon , and C. Ng . 2017 Fifth Ovarian cancer consensus conference of the gynecologic cancer intergroup: first‐line interventions. Ann. Oncol. 28:711–717. 28327917 10.1093/annonc/mdx011

[3] George, A. , S. Kaye , and S. Banerjee . 2017 Delivering widespread BRCA testing and PARP inhibition to patients with ovarian cancer. Nat. Rev. Clin. Oncol. 14:284–296. 27958297 10.1038/nrclinonc.2016.191

[4] Jayson, G. C. , R. Kerbel , L. M. Ellis , and A. L. Harris . 2016 Antiangiogenic therapy in oncology: current status and future directions. Lancet 388:518–529. 26853587 10.1016/S0140-6736(15)01088-0

[5] Ocana, A. , and A. Pandiella . 2017 Targeting oncogenic vulnerabilities in triple negative breast cancer: biological bases and ongoing clinical studies. Oncotarget 8:22218–22234. 28108739 10.18632/oncotarget.14731 PMC5400659

[6] Ocaña, A. , J. Pérez‐Peña , A. Alcaraz‐Sanabria , V. Sánchez‐Corrales , C. Nieto‐Jiménez , A. J. Templeton , B. Seruga , A. Pandiella , and E. Amir . 2016 In silico analyses identify gene‐sets, associated with clinical outcome in ovarian cancer: role of mitotic kinases. Oncotarget 7:22865–22872. 26992217 10.18632/oncotarget.8118 PMC5008407

[7] Cerami, E. , J. Gao , U. Dogrusoz , B. E. Gross , S. O. Sumer , B. A. Aksoy , A. Jacobsen , C. J. Byrne , M. L. Heuer , E. Larsson , and Y. Antipin . 2012 The cBio cancer genomics portal: an open platform for exploring multidimensional cancer genomics data. Cancer Discov. 2:401–404. 22588877 10.1158/2159-8290.CD-12-0095 PMC3956037

[8] Dominguez‐Brauer, C. , K. L. Thu , J. M. Mason , H. Blaser , M. R. Bray , and T. W. Mak . 2015 Targeting mitosis in cancer: emerging strategies. Mol. Cell 60:524–536. 26590712 10.1016/j.molcel.2015.11.006

[9] San‐Miguel, J. F. , V. T. Hungria , S. S. Yoon , M. Beksac , M. A. Dimopoulos , A. Elghandour , W. W. Jedrzejczak , A. Günther , T. N. Nakorn , N. Siritanaratkul , and P. Corradini . 2014 Panobinostat plus bortezomib and dexamethasone versus placebo plus bortezomib and dexamethasone in patients with relapsed or relapsed and refractory multiple myeloma: a multicentre, randomised, double‐blind phase 3 trial. Lancet Oncol. 15:1195–1206. 25242045 10.1016/S1470-2045(14)70440-1

[10] San Miguel, J. F. , R. Schlag , N. K. Khuageva , M. A. Dimopoulos , O. Shpilberg , M. Kropff , I. Spicka , M. T. Petrucci , A. Palumbo , O. S. Samoilova , and A. Dmoszynska . 2008 Bortezomib plus melphalan and prednisone for initial treatment of multiple myeloma. N. Engl. J. Med. 359:906–917. 18753647 10.1056/NEJMoa0801479

[11] Gallo, L. H. , J. Ko , and D. J. Donoghue . 2017 The importance of regulatory ubiquitination in cancer and metastasis. Cell Cycle 16:634–648. 28166483 10.1080/15384101.2017.1288326 PMC5397262

[12] Bajaj, S. , S. K. Alam , K. S. Roy , A. Datta , S. Nath , and S. Roychoudhury . 2016 E2 Ubiquitin‐conjugating Enzyme, UBE2C Gene, Is Reciprocally Regulated by Wild‐type and Gain‐of‐Function Mutant p53. J. Biol. Chem. 291:14231–14247. 27129209 10.1074/jbc.M116.731398 PMC4933179

[13] Palumbo, A. Jr , N. M. Da Costa , M. De Martino , R. Sepe , S. Pellecchia , V. P. L. de Sousa , P. N. Neto , C. D. Kruel , A. Bergman , L. E. Nasciutti , and A. Fusco . 2016 UBE2C is overexpressed in ESCC tissues and its abrogation attenuates the malignant phenotype of ESCC cell lines. Oncotarget 7:65876–65887. 27588470 10.18632/oncotarget.11674 PMC5323199

[14] Lin, J. , D. A. Raoof , Z. Wang , M. Y. Lin , D. G. Thomas , J. K. Greenson , T. J. Giordano , M. B. Orringer , A. C. Chang , D. G. Beer , and L. Lin . 2006 Expression and effect of inhibition of the ubiquitin‐conjugating enzyme E2C on esophageal adenocarcinoma. Neoplasia 8:1062–1071. 17217624 10.1593/neo.05832 PMC1783715

[15] Zhang, Z. , P. Liu , J. Wang , T. Gong , F. Zhang , J. Ma , and N. Han . 2015 Ubiquitin‐conjugating enzyme E2C regulates apoptosis‐dependent tumor progression of non‐small cell lung cancer via ERK pathway. Med. Oncol. 32:149. 25832867 10.1007/s12032-015-0609-8

[16] Cardenas, H. , J. Zhao , E. Vieth , K. P. Nephew , and D. Matei . 2016 EZH2 inhibition promotes epithelial‐to‐mesenchymal transition in ovarian cancer cells. Oncotarget 7:84453–84467. 27563817 10.18632/oncotarget.11497 PMC5356672

[17] Chan‐Penebre, E. , K. Armstrong , A. Drew , A. R. Grassian , I. Feldman , S. K. Knutson , K. Kuplast‐Barr , M. Roche , J. Campbell , P. Ho , and R. A. Copeland . 2017 Selective killing of SMARCA2‐ and SMARCA4‐deficient small cell carcinoma of the ovary, hypercalcemic type cells by inhibition of EZH2: in vitro and in vivo preclinical models. Mol. Cancer Ther. 16:850–860. 28292935 10.1158/1535-7163.MCT-16-0678

[18] Christofides, A. , T. Karantanos , K. Bardhan , and V. A. Boussiotis . 2016 Epigenetic regulation of cancer biology and anti‐tumor immunity by EZH2. Oncotarget 7:85624–85640. 27793053 10.18632/oncotarget.12928 PMC5356764

[19] Mazina, O. M. , M. J. Rossi , N. H. Thomaa , and A. V. Mazin . 2007 Interactions of human rad54 protein with branched DNA molecules. J. Biol. Chem. 282:21068–21080. 17545145 10.1074/jbc.M701992200

[20] Ritter, A. , M. Sanhaji , A. Friemel , S. Roth , U. Rolle , F. Louwen , and J. Yuan . 2015 Functional analysis of phosphorylation of the mitotic centromere‐associated kinesin by Aurora B kinase in human tumor cells. Cell Cycle 14:3755–3767. 26148251 10.1080/15384101.2015.1068481 PMC4825789

