# Supplementary material for: Functional transcriptomic annotation and protein–protein interaction analysis identify EZH2 and UBE2C as key upregulated proteins in ovarian cancer
Source: Cancer Med. 2018 Mar 25;7(5):1896–907. doi: 10.1002/cam4.1406 (PMC5943485; doi:10.1002/cam4.1406)
Supplement: Supplementary file 1 — Table S1. Functional classification of the deregulated genes. [file CAM4-7-1896-s001.pdf]

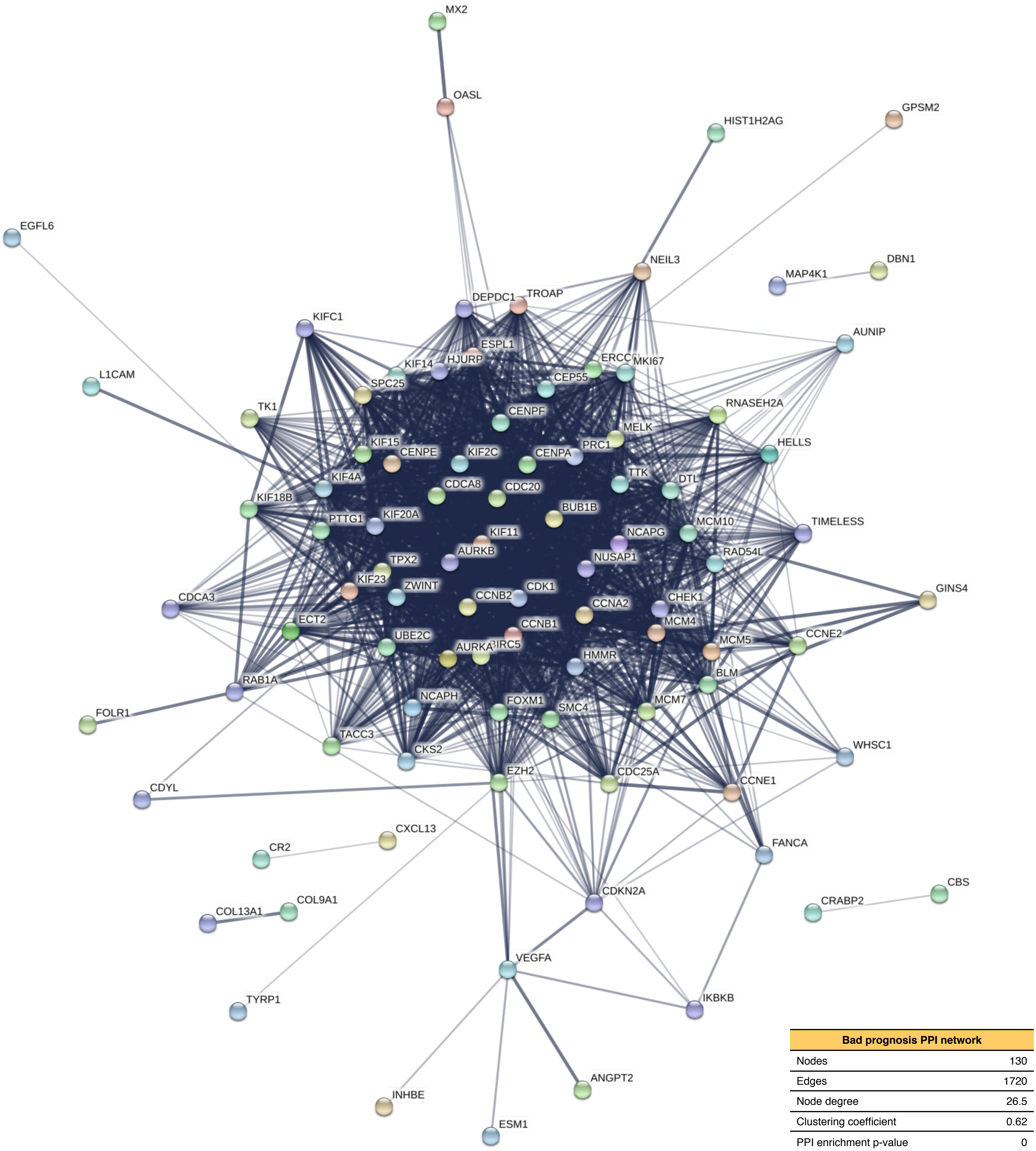

Bad prognosis PPI network	
Nodes	130
Edges	1720
Node degree	26.5
Clustering coefficient	0.62
PPI enrichment p-value	0

Supplementary Figure 1

	Angiogenesis	Apoptotic process /Cell death	Cell adhesion	Cell cycle	Cell differentiation	Cell division	Cell migration	Cell proliferation	Cellular component organization	Cellular response to EC stimuli	Chemotaxis	Development	DNA replication	Homeostasis	Inflammatory response	Inmune response	Intracellular transport	Metabolic process	Regulation of circadian rythm	Signal transduction / protein modification	Transcription regulation	Viral process
**AUNIP**						•																
**AURKA**		•		•		•				•										•		
**AURKB**				•		•				•										•	•	
**BIRC5**		•		•		•		•		•										•	•	
**BLM**						•				•			•					•			•	
**BUB1B**		•		•		•		•										•		•		
**CCNA2**				•		•						•								•	•	•
**CCNB1**				•	•	•				•		•								•		
**CCNB2**				•								•		•								
**CCNE1**				•		•														•		
**CCNE2**				•		•														•		
**CDC20**						•						•						•		•		
**CDC25A**				•		•				•										•		
**CDCA3**				•		•														•		
**CDCA8**				•		•														•		
**CDK1**		•		•		•				•										•		
**CDKN2A**	•	•	•	•																•	•	
**CDYL**					•																•	
**CENPA**						•																•
**CENPE**				•		•										•	•			•		
**CENPF**				•	•	•				•							•				•	
**CEP55**				•		•																
**CHEK1**		•		•		•				•										•	•	
**CKS2**				•																•	•	
**DEPDC1**																				•	•	
**DTL**				•									•							•		
**ECT2**		•		•	•												•	•		•		
**ERCC6L**				•		•																
**ESPL1**		•				•																
**EZH2**				•	•					•										•	•	
**FANCA**					•					•					•							
**FOXM1**				•						•								•		•	•	
**GINS4**													•									
**HELLS**		•		•		•															•	
**HJURP**				•		•														•		
**HMMR**						•												•				
**IKBKB**	•									•					•	•				•	•	
**KIF11**				•		•										•	•					
**KIF14**		•	•	•		•	•					•						•		•		
**KIF15**				•		•										•	•					
**KIF18B**				•		•																
**KIF20A**																	•					
**KIF23**				•		•										•	•					
**KIF2C**				•		•										•	•					
**KIF4A**						•										•	•					
**KIFC1**				•	•	•																
**MCM10**								•		•												
**MCM4**				•									•									
**MCM5**				•		•																
**MCM7**				•						•			•							•		
**MELK**		•		•	•													•		•		
**MKI67**				•		•				•								•				
**NCAPG**				•		•																
**NCAPH**				•		•																
**NEIL3**										•								•				
**NUSAP1**																						
**OASL**																•						
**PRC1**				•		•		•														
**PTTG1**				•	•	•				•										•	•	
**RAB1A**							•										•	•		•		
**RAD54L**						•				•												
**RNASEH2A**													•					•				
**SMC4**				•		•																
**SPC25**				•		•																
**TACC3**				•		•						•										
**TIMELESS**				•		•				•									•		•	
**TK1**													•					•		•		
**TPX2**		•				•												•		•		
**TROAP**			•																			
**TTK**				•																•		
**UBE2C**				•		•												•		•		
**VEGFA**	•	•	•		•	•		•		•	•									•	•	
**WHSC1**																					•	
**ZWINT**				•		•																

Supplementary Table 1

GENE	DRUGS
EZH2	EL1, EPZ-6438, GSK126
VEGFA	CARVEDILOL, GLICLAZIDE, MINOCYCLINE, PEGAPTANIB, 4SC-202, ABT-510, BEVACIZUMAB+RITUXIMAB, BEVASIRANIB, ENDOSTATIN (84-114)-NH2 (JKC367), LENALIDOMIDE, SEMAXANIB, SORAFENIB TOSYLATE, TAK-593, THALIDOMIDE, VANDETANIB
AURKA	AT9283, ENMD-2076, ALISERTIB, PF-03814735, PHOSPHONOTHREONINE, SNS-314, TOZASERTIB, 4-(4-METHYLPIPERAZIN-1-YL)-N-[5-(2-THIENYLACETYL)-1,5-DIHYDROPYRROLO[3,4-C]PYRAZOL-3- YL]BENZAMIDE, CISPLATINUM, N-[3-(1H-BENZIMIDAZOL-2-YL)-1H-PYRAZOL-4-YL]BENZAMIDE, PACLITAXEL, 722544-51-6, ALISERTIB SODIUM, AMG 900, AURORA KINASE INHIBITOR II, AURORA KINASE INHIBITOR III, CYC-116, DANUSERTIB, GSK-3 INHIBITOR XIII, MK-5108, MLN8054, RHO KINASE INHIBITOR IV
BIRC5	PLATINUM, TAXANE, DOCETAXEL, PACLITAXEL, TERAMEPROCOL
KIF11	3-[(5S)-1-ACETYL-3-(2-CHLOROPHENYL)-4,5-DIHYDRO-1H-PYRAZOL-5-YL]PHENOL, MONASTROL, ISPINESIB, (2S)-4-(2,5-DIFLUOROPHENYL)-N,N-DIMETHYL-2-PHENYL-2,5-DIHYDRO-1H-PYRROLE-1- CARBOXAMIDE, (2S)-4-(2,5-DIFLUOROPHENYL)-N-METHYL-2-PHENYL-N-PIPERIDIN-4-YL-2,5-DIHYDRO- 1H-PYRROLE-1-CARBOXAMIDE, (5R)-N,N-DIETHYL-5-METHYL-2-[(THIOPHEN-2-YLCARBONYL)AMINO]- 4,5,6,7-TETRAHYDRO-1-BENZOTHIOPHENE-3-CARBOXAMIDE, (5S)-5-(3-AMINOPROPYL)-3-(2,5- DIFLUOROPHENYL)-N-ETHYL-5-PHENYL-4,5-DIHYDRO-1H-PYRAZOLE-1-CARBOXAMIDE, ARRY-520, SB- 743921
UBE2C	NSC697923
CENPE	GSK-923295
MCM7	Atorvastatin
EZH2	s-adenosylhomocysteine, EL1, EPZ-6438, 3-Deazaneplanocin A (DZNep) hydrochloride, 3-Deazaneplanocin (DZNep), CPI-169, CPI-169 R-enantiomer, CPI-169 S-enantiomer, EPZ005687, GSK343, GSK503, GSK126, UNC 2400, UNC 1999, CPI-1205
AURKA	Phosphonothreonine, Barasertib (AZD1152-HQPA), AT9283, CYC116, Alisertib (MLN8237), XL228, ABT-384, AMG900, Anacardic acid, AS703569, Aurora A Inhibitor I, AURORA KINASE INHIBITOR II, AURORA KINASE INHIBITOR III, Bendamustine HCl, BI-847325, CCT129202, CCT137690, Danusertib (PHA-739358), ENMD-0276, ENMD-2076, ENMD-2076 L-(+)-Tartaric acid, GSK1070916, GSK-3 INHIBITOR XIII, Hesperadin, JNJ-7706621, KW-2449, MK-5108 (VX-689), MK-8745, MLN8054, PF-03814735, PHA-680632, R763, Reversine, RHO KINASE INHIBITOR IV, SCH-1473759, SN-314, SNS-314 Mesylate, TAK901, TC-A 2317 hydrochloride, Tozasertib (MK-0457,VX-680), ZM 447439
TTK	AZ3146, MPI-0479605, Mps1-IN-1, Mps1-IN-2, Mps1-IN-3, NMS-P715, BAY 1217389
MKI67	Fulvestrant
KIF2C	DB04395, GSK-923295, MPI-0479605, SB743921
CCNE1	ALISERTIB, DINACICLIB (SCH-727965), AMG900, BAY1000394
MELK	OTSSP167, OTSSP167 hydrochloride
CDC25A	MENADIONE, NSC 95397, NSC 663284
BIRC5	Docetaxel, Paclitaxel, LY2181308, YM155 (Sepantronium Bromide), YM-155 hydrochloride, HGS1029, SPC-3042
CDKN2A	LEE011, LY2835219, BAY1000394, PD-0332991

HMMR	HYALURONIC ACID
VEGFA	4SC-202, ABT-510, AE-941, Aflibercept, Atorvastatin, Avastin, Bevacizumab, Bevasiranib, Capecitabine, Carboplatin, Carvedilol, Cisplatin, Combretastatin a4, Cyclophosphamide, Dalteparin, Docetaxel, Enalapril, Endostatins, EW-A-401, Fluorouracil, Gliclazide, Heparin, Irinotecan, Lenalidomide, Lenalidomide, Leucovorin, Minocycline, Nintedanib (BIBF 1120), Oxaliplatin, Pegaptanib, Pyroglutamic acid, Ranibizumab, Regorafenib, SB-509, Sildenafil, Simvastatin, Sorafenib, Sorafenib Tosylate, Sunitinib, taxanes, Thalidomide, Tris, Vandetanib, VEGF GENE THERAPY, VEGF-121, VEGF-AS, VEGLIN, Zaltrap, ziv-aflibercept
BLM	Bleomycin, Dacarbazine, Doxil, Doxorubicin, Lenograstim, rituximab, Vinblastine, Vincristine, Phosphoric acid, Water, Adjuvants, Immunologic, Alkylating Agents, Anti-Bacterial Agents, Antibiotics, Antitubercular, Anti-Infective Agents, Antimitotic Agents, Antineoplastic Agents, Alkylating, Antineoplastic Agents, Phytogenic, Antirheumatic Agents, Antiviral Agents, Interferon-alpha, interferons, Topoisomerase Inhibitors, ML216, Magnesium
RAD54L	RUCAPARIB, Poly(ADP-ribose) Polymerase Inhibitors
KIF4A	SPINESIB (SB-715992), SB-743921
IKBKB	Acetylcysteine, ACHP, Amlexanox, Andrographolide, Arsenic trioxide, Aspirin, Auranofin, Bardoxolone methyl, Bay 11-7085, Bay 11-7821(BAY 11-7082), Bay 65-1942 free base, Bay 65-1942 HCl salt, Bay 65-1942 R form, BMS-345541, BMS-345541(free base), Choline Fenofibrate, Honokiol, IKK-16 (IKK Inhibitor VII), IKK-2 INHIBITOR IV, IKK-2 inhibitor VIII, IMD 0354, LY2409881, Mesalamine, Mesalazine, ML 120B dihydrochloride, MLN0415, MLN-0415, MLN120B, PF 184, PS 1145 dihydrochloride, SC-514, Sulfasalazine, Sumatriptan, Tanshinone IIA, TPCA-1, Trisenox, WS6
TK1	5-Thymidylic acid, Deoxyuridine, Dithioerythritol, Floxuridine, Thymidine, Thymidine-5'-Triphosphate, Trifluridine, Zidovudine
FANCA	RUCAPARIB, Poly(ADP-ribose) Polymerase Inhibitors

Gene Name	PFR		OS	
	HR	*p*-value	HR	*p*-value
EZH2	*3,63 (1,93 – 6,8)*	*1,80E-005*	*3,29 (1,38 – 7,88)*	*0,0046*
RAD54L	*2,85 (1,53 – 5,32)*	*,00058*	*2,03 (0,9 – 4,55)*	*0,081*
AURKA	*2,95 (1,6 – 5,45)*	*3,00E-004*	*2,34 (1,04 – 5,26)*	*0,034*
KIF2C	*2,43 (1,33 – 4,44)*	*0,003*	*2,18 (0,97 – 4,91)*	*0,055*
BIRC5	*2,85 (1,53 – 5,31)*	*0,00058*	*1,15 (0,53 – 2,49)*	*0,72*
UBE2C	*3,03 (1,62 – 5,66)*	*0,00026*	*2,3 (1,02 – 5,17)*	*0,038*
BLM	*1,99 (1,1 – 3,59)*	*0,02*	*2,45 (1,06 – 5,68)*	*0,03*
CHEK1	*4,29 (1,84 – 10,01)*	*0,00025*	*6,83 (1,54 – 30,29)*	*0,0033*
MKI67	*1,94 (1,07 – 3,51)*	*0,026*	*2,85 (1,23 – 6,61)*	*0,011*
MCM7	*2,8 (1,5 – 5,23)*	*0,00072*	*2,09 (0,93 – 4,71)*	*0,068*
KIF4A	*2,82 (1,51 – 5,27)*	*0,00066*	*2,09 (0,93 – 4,71)*	*0,07*
CDK1	*1,8 (1,01 – 3,23)*	*0,045*	*1,31 (0,61 – 2,85)*	*0,49*
TTK	*2,52 (1,38 – 4,61)*	*0,0019*	*1,74 (0,79 – 3,83)*	*0,17*
MELK	*1,95 (1,08 – 3,5)*	*0,023*	*1,31 (0,6 – 2,83)*	*0,5*
KIF15	*2,88 (1,54 – 5,38)*	*0,00053*	*1,96 (0,88 – 4,33)*	*0,091*
CENPE	*2,55 (1,36 – 4,75)*	*0,0024*	*1,68 (0,75 – 3,78)*	*0,2*
AURKB	*1,9 (1,06 – 3,4)*	*0,03*	*2,78 (1,2 – 6,41)*	*0,012*
KIF11	*2,73 (1,48 – 5,03)*	*0,00083*	*2,06 (0,92 – 4,62)*	*0,074*
