# Supplementary material for: Functional transcriptomic annotation and protein–protein interaction analysis identify EZH2 and UBE2C as key upregulated proteins in ovarian cancer
Source: Cancer Med. 2018 Mar 25;7(5):1896–907. doi: 10.1002/cam4.1406 (PMC5943485; doi:10.1002/cam4.1406)
Supplement: Supplementary file 2 — Table S2. List of potentially druggable genes. [file CAM4-7-1896-s002.doc]

|  | **Angiogenesis** | **Apoptotic process /Cell death** | **Cell adhesion** | **Cell cycle** | **Cell differentiation** | **Cell division** | **Cell migration** | **Cell proliferation** | **Cellular component organization** | **Cellular response to EC stimuli** | **Chemotaxis** | **Development** | **DNA replication** | **Homeostasis** | **Inflammatory response** | **Inmune response** | **Intracellular transport** | **Metabolic process** | **Regulation of circadian rythm** | **Signal transduction / protein modification** | **Transcription regulation** | **Viral process** |
| --- | --- | --- | --- | --- | --- | --- | --- | --- | --- | --- | --- | --- | --- | --- | --- | --- | --- | --- | --- | --- | --- | --- |
| **AUNIP** |  |  |  |  |  | **●** |  |  |  |  |  |  |  |  |  |  |  |  |  |  |  |  |
| **AURKA** |  | **●** |  | **●** |  | **●** |  |  |  | **●** |  |  |  |  |  |  |  |  |  | **●** |  |  |
| **AURKB** |  |  |  | **●** |  | **●** |  |  |  | **●** |  |  |  |  |  |  |  |  |  | **●** | **●** |  |
| **BIRC5** |  | **●** |  | **●** |  | **●** |  | **●** |  | **●** |  |  |  |  |  |  |  |  |  | **●** | **●** |  |
| **BLM** |  |  |  |  |  | **●** |  |  |  | **●** |  |  | **●** |  |  |  |  | **●** |  |  | **●** |  |
| **BUB1B** |  | **●** |  | **●** |  | **●** |  | **●** |  |  |  |  |  |  |  |  |  | **●** |  | **●** |  |  |
| **CCNA2** |  |  |  | **●** |  | **●** |  |  |  |  |  | **●** |  |  |  |  |  |  |  | **●** | **●** | **●** |
| **CCNB1** |  |  |  | **●** | **●** | **●** |  |  |  | **●** |  | **●** |  |  |  |  |  |  |  | **●** |  |  |
| **CCNB2** |  |  |  | **●** |  |  |  |  |  |  |  | **●** |  | **●** |  |  |  |  |  |  |  |  |
| **CCNE1** |  |  |  | **●** |  | **●** |  |  |  |  |  |  |  |  |  |  |  |  |  | **●** |  |  |
| **CCNE2** |  |  |  | **●** |  | **●** |  |  |  |  |  |  |  |  |  |  |  |  |  | **●** |  |  |
| **CDC20** |  |  |  |  |  | **●** |  |  |  |  |  | **●** |  |  |  |  |  | **●** |  | **●** |  |  |
| **CDC25A** |  |  |  | **●** |  | **●** |  |  |  | **●** |  |  |  |  |  |  |  |  |  | **●** |  |  |
| **CDCA3** |  |  |  | **●** |  | **●** |  |  |  |  |  |  |  |  |  |  |  |  |  | **●** |  |  |
| **CDCA8** |  |  |  | **●** |  | **●** |  |  |  |  |  |  |  |  |  |  |  |  |  | **●** |  |  |
| **CDK1** |  | **●** |  | **●** |  | **●** |  |  |  | **●** |  |  |  |  |  |  |  |  |  | **●** |  |  |
| **CDKN2A** |  | **●** | **●** | **●** |  |  |  |  |  |  |  |  |  |  |  |  |  |  |  | **●** | **●** |  |
| **CDYL** |  |  |  |  | **●** |  |  |  |  |  |  |  |  |  |  |  |  |  |  |  | **●** |  |
| **CENPA** |  |  |  |  |  | **●** |  |  |  |  |  |  |  |  |  |  |  |  |  |  |  | **●** |
| **CENPE** |  |  |  | **●** |  | **●** |  |  |  |  |  |  |  |  |  | **●** | **●** |  |  | **●** |  |  |
| **CENPF** |  |  |  | **●** | **●** | **●** |  |  |  | **●** |  |  |  |  |  |  | **●** |  |  |  | **●** |  |
| **CEP55** |  |  |  | **●** |  | **●** |  |  |  |  |  |  |  |  |  |  |  |  |  |  |  |  |
| **CHEK1** |  | **●** |  | **●** |  | **●** |  |  |  | **●** |  |  |  |  |  |  |  |  |  | **●** | **●** |  |
| **CKS2** |  |  |  | **●** |  |  |  |  |  |  |  |  |  |  |  |  |  |  |  | **●** | **●** |  |
| **DEPDC1** |  |  |  |  |  |  |  |  |  |  |  |  |  |  |  |  |  |  |  | **●** | **●** |  |
| **DTL** |  |  |  | **●** |  |  |  |  |  |  |  |  | **●** |  |  |  |  |  |  | **●** |  |  |
| **ECT2** |  | **●** |  | **●** | **●** |  |  |  |  |  |  |  |  |  |  |  | **●** | **●** |  | **●** |  |  |
| **ERCC6L** |  |  |  | **●** |  | **●** |  |  |  |  |  |  |  |  |  |  |  |  |  |  |  |  |
| **ESPL1** |  | **●** |  |  |  | **●** |  |  |  |  |  |  |  |  |  |  |  |  |  |  |  |  |
| **EZH2** |  |  |  | **●** | **●** |  |  |  |  | **●** |  |  |  |  |  |  |  |  |  | **●** | **●** |  |
| **FANCA** |  |  |  |  | **●** |  |  |  |  | **●** |  |  |  |  | **●** |  |  |  |  |  |  |  |
| **FOXM1** |  |  |  | **●** |  |  |  |  |  | **●** |  |  |  |  |  |  |  | **●** |  | **●** | **●** |  |
| **GINS4** |  |  |  |  |  |  |  |  |  |  |  |  | **●** |  |  |  |  |  |  |  |  |  |
| **HELLS** |  | **●** |  | **●** |  | **●** |  |  |  |  |  |  |  |  |  |  |  |  |  |  | **●** |  |
| **HJURP** |  |  |  | **●** |  | **●** |  |  |  |  |  |  |  |  |  |  |  |  |  | **●** |  |  |
| **HMMR** |  |  |  |  |  | **●** |  |  |  |  |  |  |  |  |  |  |  | **●** |  |  |  |  |
| **IKBKB** | **●** |  |  |  |  |  |  |  |  | **●** |  |  |  |  | **●** | **●** |  |  |  | **●** | **●** |  |
| **KIF11** |  |  |  | **●** |  | **●** |  |  |  |  |  |  |  |  |  | **●** | **●** |  |  |  |  |  |
| **KIF14** |  | **●** | **●** | **●** |  | **●** | **●** |  |  |  |  | **●** |  |  |  |  |  | **●** |  | **●** |  |  |
| **KIF15** |  |  |  | **●** |  | **●** |  |  |  |  |  |  |  |  |  | **●** | **●** |  |  |  |  |  |
| **KIF18B** |  |  |  | **●** |  | **●** |  |  |  |  |  |  |  |  |  |  |  |  |  |  |  |  |
| **KIF20A** |  |  |  |  |  |  |  |  |  |  |  |  |  |  |  |  | **●** |  |  |  |  |  |
| **KIF23** |  |  |  | **●** |  | **●** |  |  |  |  |  |  |  |  |  | **●** | **●** |  |  |  |  |  |
| **KIF2C** |  |  |  | **●** |  | **●** |  |  |  |  |  |  |  |  |  | **●** | **●** |  |  |  |  |  |
| **KIF4A** |  |  |  |  |  | **●** |  |  |  |  |  |  |  |  |  | **●** | **●** |  |  |  |  |  |
| **KIFC1** |  |  |  | **●** | **●** | **●** |  |  |  |  |  |  |  |  |  |  |  |  |  |  |  |  |
| **MCM10** |  |  |  |  |  |  |  | **●** |  | **●** |  |  |  |  |  |  |  |  |  |  |  |  |
| **MCM4** |  |  |  | **●** |  |  |  |  |  |  |  |  | **●** |  |  |  |  |  |  |  |  |  |
| **MCM5** |  |  |  | **●** |  | **●** |  |  |  |  |  |  |  |  |  |  |  |  |  |  |  |  |
| **MCM7** |  |  |  | **●** |  |  |  |  |  | **●** |  |  | **●** |  |  |  |  |  |  | **●** |  |  |
| **MELK** |  | **●** |  | **●** | **●** |  |  |  |  |  |  |  |  |  |  |  |  | **●** |  | **●** |  |  |
| **MKI67** |  |  |  | **●** |  | **●** |  |  |  | **●** |  |  |  |  |  |  |  | **●** |  |  |  |  |
| **NCAPG** |  |  |  | **●** |  | **●** |  |  |  |  |  |  |  |  |  |  |  |  |  |  |  |  |
| **NCAPH** |  |  |  | **●** |  | **●** |  |  |  |  |  |  |  |  |  |  |  |  |  |  |  |  |
| **NEIL3** |  |  |  |  |  |  |  |  |  | **●** |  |  |  |  |  |  |  | **●** |  |  |  |  |
| **NUSAP1** |  |  |  |  |  |  |  |  |  |  |  |  |  |  |  |  |  |  |  |  |  |  |
| **OASL** |  |  |  |  |  |  |  |  |  |  |  |  |  |  |  | **●** |  |  |  |  |  |  |
| **PRC1** |  |  |  | **●** |  | **●** |  | **●** |  |  |  |  |  |  |  |  |  |  |  |  |  |  |
| **PTTG1** |  |  |  | **●** | **●** | **●** |  |  |  | **●** |  |  |  |  |  |  |  |  |  | **●** | **●** |  |
| **RAB1A** |  |  |  |  |  |  | **●** |  |  |  |  |  |  |  |  |  | **●** | **●** |  | **●** |  |  |
| **RAD54L** |  |  |  |  |  | **●** |  |  |  | **●** |  |  |  |  |  |  |  |  |  |  |  |  |
| **RNASEH2A** |  |  |  |  |  |  |  |  |  |  |  |  | **●** |  |  |  |  | **●** |  |  |  |  |
| **SMC4** |  |  |  | **●** |  | **●** |  |  |  |  |  |  |  |  |  |  |  |  |  |  |  |  |
| **SPC25** |  |  |  | **●** |  | **●** |  |  |  |  |  |  |  |  |  |  |  |  |  |  |  |  |
| **TACC3** |  |  |  | **●** |  | **●** |  |  |  |  |  | **●** |  |  |  |  |  |  |  |  |  |  |
| **TIMELESS** |  |  |  | **●** |  | **●** |  |  |  | **●** |  |  |  |  |  |  |  |  | **●** |  | **●** |  |
| **TK1** |  |  |  |  |  |  |  |  |  |  |  |  | **●** |  |  |  |  | **●** |  | **●** |  |  |
| **TPX2** |  | **●** |  |  |  | **●** |  |  |  |  |  |  |  |  |  |  |  | **●** |  | **●** |  |  |
| **TROAP** |  |  | **●** |  |  |  |  |  |  |  |  |  |  |  |  |  |  |  |  |  |  |  |
| **TTK** |  |  |  | **●** |  |  |  |  |  |  |  |  |  |  |  |  |  |  |  | **●** |  |  |
| **UBE2C** |  |  |  | **●** |  | **●** |  |  |  |  |  |  |  |  |  |  |  | **●** |  | **●** |  |  |
| **VEGFA** | **●** | **●** | **●** |  | **●** | **●** |  | **●** |  | **●** | **●** |  |  |  |  |  |  |  |  | **●** | **●** |  |
| **WHSC1** |  |  |  |  |  |  |  |  |  |  |  |  |  |  |  |  |  |  |  |  | **●** |  |
| **ZWINT** |  |  |  | **●** |  | **●** |  |  |  |  |  |  |  |  |  |  |  |  |  |  |  |  |
