# Supplementary material for: Functional transcriptomic annotation and protein–protein interaction analysis identify EZH2 and UBE2C as key upregulated proteins in ovarian cancer
Source: Cancer Med. 2018 Mar 25;7(5):1896–907. doi: 10.1002/cam4.1406 (PMC5943485; doi:10.1002/cam4.1406)
Supplement: Supplementary file 3 — Table S3. Association with progression free survival (PFS) and overall survival (OS) of the identified hub proteins. [file CAM4-7-1896-s003.docx]

| **GENE** | **DRUGS** |
| --- | --- |
| KIF15 | Kif15-IN-1, Kif15-IN-2, SB743921 HCl |
| CDK1 | Deferasirox, Alsterpaullone, Hymenialdisine, Indirubin-3'-Monoxime, Nocodazole, Olomoucine, SU9516, Flavopiridol, AT7519, (R)-DRF053 dihydrochloride, ALOISINE A, ALSTERPAULLONE 2-CYANOETHYL, Alvocidib, Aminopurvalanol A, AT7519 Hydrochloride, AT7519 trifluoroacetate, AURORA KINASE/CDK INHIBITOR, AZD-5438, AZD-5597, BAY1000394, BMS265246, BOHEMINE, BS-181 HCl, CDK inhibitor II, CDK1 INHIBITOR, CDK1 INHIBITOR (CGP74514A), CDK1/2 INHIBITOR III, CDK1/5 INHIBITOR, CDK2 INHIBITOR III, CDK2 INHIBITOR IV, CDK4 inhibitor, CGP60474, COMPOUND 52 [PMID:9677190], Dinaciclib (SCH-727965), Flavopiridol hydrochloride, GSK-3 INHIBITOR IX, JNJ-7706621, Kenpaullone, LDC000067, NSC 693868, Nu 6027, NVP-LCQ195, P276-00, PF 4800567 hydrochloride, PHA-767491, PHA-793887, PHA-848125, Purvalanol B, R547, radiotherapy, RGB-286638, Ro 3306, RONICICLIB, SCH727965, Senexin A, SNS-032 (BMS-387032), TMCB |
| KIF11 | Monastrol, GSK-923295, 4SC-205, ALN-VSP, ARQ-621, ARRY-520, AZ 3146, AZD4877, BAY 1217389, Ispinesib (SB-715992), LY-2523355, MK0731, MPI-0479605, Paprotrain, SB-743921 |
| CHEK1 | CHIR-124, PF-477736, MK-8776 (SCH 900776), LY2603618, AZD7762, CCT245737, SAR-020106, XL844, LY2606368, TCS 2312, isogranulatimide, CCT244747, Chk1 and MK2 Inhibitors set, NSC 109555 ditosylate, SB-218078 |
| AURKB | Hesperidin, AT9283, CYC116, ABT-384, AMG 900, Anacardic acid, AS703569, Aurora A Inhibitor I, AURORA KINASE INHIBITOR II, Barasertib (AZD1152-HQPA), BI847325, CCT129202, CCT137690, Danusertib (PHA-739358), ENMD-2076, ENMD-2076 L-(+)-Tartaric acid, GSK1070916, Hesperadin, JNJ-7706621, Magnesium, MK-5108 (VX-689), MK-8745, MLN8054, PF-03814735, PHA-680632, R763, Reversine, SCH-1473759, SN-314, SNS-314 Mesylate, SU6656, TAK-901, TC-A 2317 hydrochloride, Tozasertib (MK-0457,VX-680), ZM 447439 |
| UBE2C | NSC697923 |
| CENPE | GSK-923295 |
| MCM7 | Atorvastatin |
| EZH2 | s-adenosylhomocysteine, EL1, EPZ-6438, 3-Deazaneplanocin A (DZNep) hydrochloride, 3-Deazaneplanocin (DZNep), CPI-169, CPI-169 R-enantiomer, CPI-169 S-enantiomer, EPZ005687, GSK343, GSK503, GSK126, UNC 2400, UNC 1999, CPI-1205 |
| AURKA | Phosphonothreonine, Barasertib (AZD1152-HQPA), AT9283, CYC116, Alisertib (MLN8237), XL228, ABT-384, AMG900, Anacardic acid, AS703569, Aurora A Inhibitor I, AURORA KINASE INHIBITOR II, AURORA KINASE INHIBITOR III, Bendamustine HCl, BI-847325, CCT129202, CCT137690, Danusertib (PHA-739358), ENMD-0276, ENMD-2076, ENMD-2076 L-(+)-Tartaric acid, GSK1070916, GSK-3 INHIBITOR XIII, Hesperadin, JNJ-7706621, KW-2449, MK-5108 (VX-689), MK-8745, MLN8054, PF-03814735, PHA-680632, R763, Reversine, RHO KINASE INHIBITOR IV, SCH-1473759, SN-314, SNS-314 Mesylate, TAK901, TC-A 2317 hydrochloride, Tozasertib (MK-0457,VX-680), ZM 447439 |
| TTK | AZ3146, MPI-0479605, Mps1-IN-1, Mps1-IN-2, Mps1-IN-3, NMS-P715, BAY 1217389 |
| MKI67 | Fulvestrant |
| KIF2C | DB04395, GSK-923295, MPI-0479605, SB743921 |
| CCNE1 | ALISERTIB, DINACICLIB (SCH-727965), AMG900, BAY1000394 |
| MELK | OTSSP167, OTSSP167 hydrochloride |
| CDC25A | MENADIONE, NSC 95397, NSC 663284 |
| BIRC5 | Docetaxel, Paclitaxel, LY2181308, YM155 (Sepantronium Bromide), YM-155 hydrochloride, HGS1029, SPC-3042 |
| CDKN2A | LEE011, LY2835219, BAY1000394, PD-0332991 |
| HMMR | HYALURONIC ACID |
| VEGFA | 4SC-202, ABT-510, AE-941, Aflibercept, Atorvastatin, Avastin, Bevacizumab, Bevasiranib, Capecitabine, Carboplatin, Carvedilol, Cisplatin, Combretastatin a4, Cyclophosphamide, Dalteparin, Docetaxel, Enalapril, Endostatins, EW-A-401, Fluorouracil, Gliclazide, Heparin, Irinotecan, Lenalidomide, Lenalidomide, Leucovorin, Minocycline, Nintedanib (BIBF 1120), Oxaliplatin, Pegaptanib, Pyroglutamic acid, Ranibizumab, Regorafenib, SB-509, Sildenafil, Simvastatin, Sorafenib, Sorafenib Tosylate, Sunitinib, taxanes, Thalidomide, Tris, Vandetanib, VEGF GENE THERAPY, VEGF-121, VEGF-AS, VEGLIN, Zaltrap, ziv-aflibercept |
| BLM | Bleomycin, Dacarbazine, Doxil, Doxorubicin, Lenograstim, rituximab, Vinblastine, Vincristine, Phosphoric acid, Water, Adjuvants, Immunologic, Alkylating Agents, Anti-Bacterial Agents, Antibiotics, Antitubercular, Anti-Infective Agents, Antimitotic Agents, Antineoplastic Agents, Alkylating, Antineoplastic Agents, Phytogenic, Antirheumatic Agents, Antiviral Agents, Interferon-alpha, interferons, Topoisomerase Inhibitors, ML216, Magnesium |
| RAD54L | RUCAPARIB, Poly(ADP-ribose) Polymerase Inhibitors |
| KIF4A | ISPINESIB (SB-715992), SB-743921 |
| IKBKB | Acetylcysteine, ACHP, Amlexanox, Andrographolide, Arsenic trioxide, Aspirin, Auranofin, Bardoxolone methyl, Bay 11-7085, Bay 11-7821(BAY 11-7082), Bay 65-1942 free base, Bay 65-1942 HCl salt, Bay 65-1942 R form, BMS-345541, BMS-345541(free base), Choline Fenofibrate, Honokiol, IKK-16 (IKK Inhibitor VII), IKK-2 INHIBITOR IV, IKK-2 inhibitor VIII, IMD 0354, LY2409881, Mesalamine, Mesalazine, ML 120B dihydrochloride, MLN0415, MLN-0415, MLN120B, PF 184, PS 1145 dihydrochloride, SC-514, Sulfasalazine, Sumatriptan, Tanshinone IIA, TPCA-1, Trisenox, WS6 |
| TK1 | 5-Thymidylic acid, Deoxyuridine, Dithioerythritol, Floxuridine, Thymidine, Thymidine-5'-Triphosphate, Trifluridine, Zidovudine |
| FANCA | RUCAPARIB, Poly(ADP-ribose) Polymerase Inhibitors |
