# Supplementary material for: Functional transcriptomic annotation and protein–protein interaction analysis identify EZH2 and UBE2C as key upregulated proteins in ovarian cancer
Source: Cancer Med. 2018 Mar 25;7(5):1896–907. doi: 10.1002/cam4.1406 (PMC5943485; doi:10.1002/cam4.1406)
Supplement: Supplementary file 4 — Figure S1. Protein‐protein interaction network of the 130 deregulated genes associated with detrimental prognosis. [file CAM4-7-1896-s004.docx]

| **Gene Name** | **PFR** | | **OS** | |
| --- | --- | --- | --- | --- |
|  | **HR** | ***p-*value** | **HR** | ***p-*value** |
| *EZH2* | 3,63 (1,93 – 6,8) | 1,80E-005 | 3,29 (1,38 – 7,88) | 0,0046 |
| *RAD54L* | 2,85 (1,53 – 5,32) | 0,00058 | 2,03 (0,9 – 4,55) | 0,081 |
| *AURKA* | 2,95 (1,6 – 5,45) | 3,00E-004 | 2,34 (1,04 – 5,26) | 0,034 |
| *KIF2C* | 2,43 (1,33 – 4,44) | 0,003 | 2,18 (0,97 – 4,91) | 0,055 |
| *BIRC5* | 2,85 (1,53 – 5,31) | 0,00058 | 1,15 (0,53 – 2,49) | 0,72 |
| *UBE2C* | 3,03 (1,62 – 5,66) | 0,00026 | 2,3 (1,02 – 5,17) | 0,038 |
| *BLM* | 1,99 (1,1 – 3,59) | 0,02 | 2,45 (1,06 – 5,68) | 0,03 |
| *CHEK1* | 4,29 (1,84 – 10,01) | 0,00025 | 6,83 (1,54 – 30,29) | 0,0033 |
| *MKI67* | 1,94 (1,07 – 3,51) | 0,026 | 2,85 (1,23 – 6,61) | 0,011 |
| *MCM7* | 2,8 (1,5 – 5,23) | 0,00072 | 2,09 (0,93 – 4,71) | 0,068 |
| *KIF4A* | 2,82 (1,51 – 5,27) | 0,00066 | 2,09 (0,93 – 4,71) | 0,07 |
| *CDK1* | 1,8 (1,01 – 3,23) | 0,045 | 1,31 (0,61 – 2,85) | 0,49 |
| *TTK* | 2,52 (1,38 – 4,61) | 0,0019 | 1,74 (0,79 – 3,83) | 0,17 |
| *MELK* | 1,95 (1,08 – 3,5) | 0,023 | 1,31 (0,6 – 2,83) | 0,5 |
| *KIF15* | 2,88 (1,54 – 5,38) | 0,00053 | 1,96 (0,88 – 4,33) | 0,091 |
| *CENPE* | 2,55 (1,36 – 4,75) | 0,0024 | 1,68 (0,75 – 3,78) | 0,2 |
| *AURKB* | 1,9 (1,06 – 3,4) | 0,03 | 2,78 (1,2 – 6,41) | 0,012 |
| *KIF11* | 2,73 (1,48 – 5,03) | 0,00083 | 2,06 (0,92 – 4,62) | 0,074 |
