# Supplementary material for: Functional transcriptomic annotation and protein–protein interaction analysis identify EZH2 and UBE2C as key upregulated proteins in ovarian cancer
Source: Cancer Med. 2018 Mar 25;7(5):1896–907. doi: 10.1002/cam4.1406 (PMC5943485; doi:10.1002/cam4.1406)
Supplement: Supplementary file 6 [file CAM4-7-1896-s006.docx]

**Table S1. Functional classification of the deregulated genes.**

**Table S2. List of potentially druggable genes.**

**Table S3. Association with progression free survival (PFS) and overall survival (OS) of the identified hub proteins.**

**Figure S1. Protein-protein interaction network of the 130** **deregulated genes associated with detrimental prognosis.**
